# Lichen sclerosus of the oral mucosa: a hidden phenomenon

**DOI:** 10.3389/froh.2024.1428008

**Published:** 2024-07-03

**Authors:** A. Paganelli, V. D. Mandel, L. Contu, A. Motolese

**Affiliations:** ^1^ Dermatology Unit, Reggio Emilia Research Hospital—Arcispedale Santa Maria Nuova, AUSL-IRCCS of Reggio Emilia, Reggio Emilia, Italy; ^2^ Porphyria and Rare Diseases Unit, San Gallicano Dermatological Institute—IRCCS, Rome, Italy

**Keywords:** lichen sclerosus, oral mucosa, lip, Inflammation, dermatology

## Abstract

Oral lichen sclerosus (OLS) represents a diagnostic challenge even for expert dermatologists due to its rarity and subtle clinical manifestations. Only few cases have been reported in literature to date. OLS typically presents with whitish macules in the oral cavity. Histopathological examination remains crucial for definitive diagnosis, with characteristic features including epithelial atrophy, subepithelial hyalinization, loss of elastic fibers, and lymphocytic infiltration. Management strategies vary depending on lesion size and symptomatic presentation, with topical or intralesional corticosteroids being the most commonly used treatment modalities. Long-term monitoring is recommended due to the potential for malignant transformation, although no cases have been reported to date. Greater awareness and understanding of OLS are essential for accurate diagnosis and effective management. Based on these findings, we recommend performing an accurate evaluation of the oral mucosa, especially when dealing with patients affected by genital or extragenital lichen sclerosus (LS). Moreover, we emphasize the importance of multidisciplinary collaboration between dermatologists and other specialists of oral disorders, such as dentists. This short review briefly summarizes available data on OLS, highlighting its diverse clinical presentations and diagnostic challenges. Despite its infrequent occurrence, OLS should be considered in the differential diagnosis of white macules in the oral cavity.

## Introduction

Lichen sclerosus (LS) is a chronic inflammatory condition affecting the mucocutaneous tissues with predominant involvement of the anogenital region. However, extragenital manifestations are not uncommon (1). It usually presents as thin, whitish patches with “cigarette paper” surface or with a smooth, shiny appearance. Extragenital LS often poses a diagnostic challenge due to its potential clinical similarities with other dermatoses, especially morphea (2–4). A skin biopsy is often required to confirm the diagnosis.

LS has for long been thought to be more prevalent in post-menopausal women. However, recent data suggest LS being more likely underreported in the pediatric population and in men than traditionally believed (5).

Pruritus, or severe itching, is a hallmark symptom, which can lead to complications like fissures, bleeding, and discomfort during sexual activity (6). As a consequence, LS can significantly impact patients’ quality of life and mental well-being (7).

Though its exact cause is unclear, autoimmune, hormonal and genetic factors are believed to play a role in LS pathogenesis (8–10). Treatment typically involves topical corticosteroids and emollients, but conventional therapies often offer limited success, and complete remission is an uncommon event (11, 12).

While involvement of the oral mucosa is exceptionally rare, it can sometimes be the sole affected area. Oral lichen sclerosus (OLS) is infrequently described in scientific literature, with only few reports of histopathologically confirmed cases being published.

The aim of the present review is to shed light on the nature of OLS, offering valuable insights into its clinical presentation, diagnostic challenges, and therapeutic considerations.

### Clinical presentation

**Figure 1:**
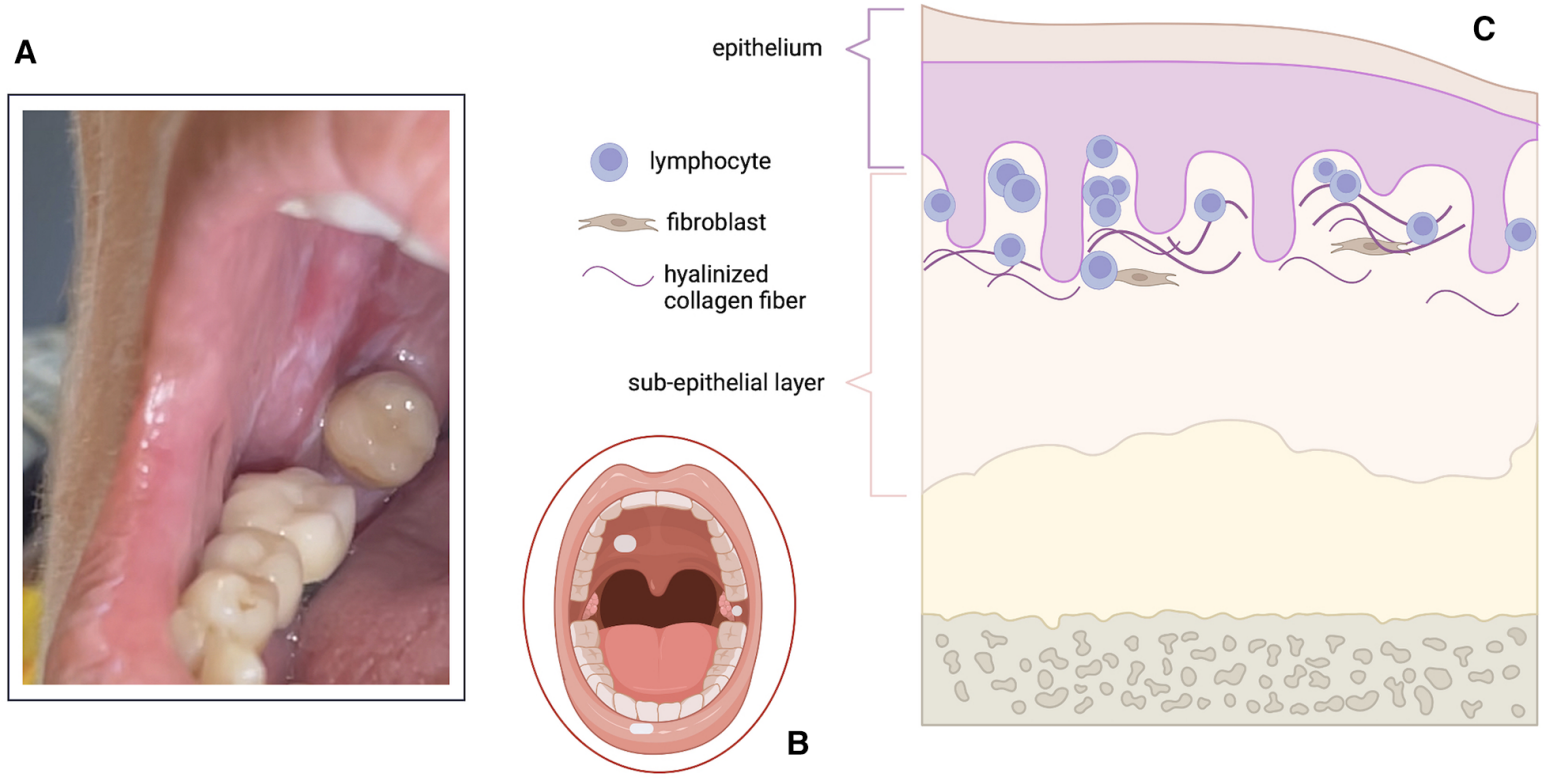
Clinical (**A,B**) and histopathological (**C**) features of oral lichen sclerosus (OLS). (**A**) Clinical image of porcelain/ivory-like whitish macules in the oral cavity. (**B**) Schematic representation of the most common locations of OLS: palatal mucosa, gingiva and lower lip. (**C**) Graphical representation of histopathological changes in OLS: sclerosis confined to the superficial sub-epithelial layers (either papillary layer or in the submucosa); lymphocytic infiltration is also present at the dermal-epidermal junction and/or at the mucosal-submucosal interface. Created with Biorender.com.

From a clinical point of view, OLS typically manifests as whitish macules, papules, or plaques, affecting the oral mucosa. Notably, lip LS lesions are often described as having vitiligoid appearance (13). Size is highly variable, ranging from few millimeters up to several centimeters (Figure 1A). Common sites of involvement in the oral cavity encompass the labial or palatal mucosa, lips (especially the lower lip), and gingiva (14). These lesions are frequently solitary; when multiple lesions are present, the generally exhibit asymmetrical distribution.

While vulvar LS commonly brings forth symptoms such as pruritus, dyspareunia, and dysuria, OLS often remains asymptomatic. No accompanying symptoms are documented in approximately 70% of cases (13). Nevertheless, individuals with OLS may experience sporadic pain, soreness, burning, and a sensation of tightness in the mouth.

It is noteworthy that over half of individuals with OLS exhibit extraoral manifestations, especially in the genital area, underscoring the importance of multidisciplinary care involving both dermatologists and gynecologists (15).

### Epidemiology

OLS represents a rarely encountered entity in clinical practice, with only limited series of confirmed cases documented in the literature to date.

A comprehensive review of these documented cases, combined with previously reported instances, sheds light on several noteworthy epidemiological trends. Notably, there appears to be a slight inclination towards women in the prevalence of OLS cases, with a male to female ratio of approximately 1.6:1 (14). However, contrasting data are currently available with regards to the age of onset. Several studies demonstrated the potential of OLS to affect individuals across all age groups, indicating no particular age predilection (15).

However, it is particularly interesting to observe that other authors suggested OLS to be predominantly diagnosed during a specific age range, with approximately 46% of cases being identified between the ages of 10–29 years (14). This observation suggests a potential clustering of OLS diagnoses within this demographic, warranting further exploration into the underlying factors contributing to this age distribution.

### Histopathology

In the realm of OLS, the typical histopathological features encompass subepithelial hyalinization, loss of elastic fibers, and a band-like inflammatory infiltrate predominantly comprised of mononuclear cells (15) (Figure 1B). Further characterization of OLS through histopathological analysis reveals additional key features, including epithelial atrophy, occasional hyperkeratosis, basal cell hydropic degeneration, lamina propria hyalinization, lymphocytic infiltration beneath the hyalinized zone, and a reduction in elastin content (14). Notably, variations in histopathological descriptions of lichen sclerosus have been observed, particularly concerning the localization of the inflammatory infiltrate in relation to the dermo-epidermal junction (16), with this variability potentially being influenced by the duration of the disease process.

While clinical differentiation between LS and lichen planus affecting the skin is generally straightforward, distinguishing between these conditions at mucosal sites often proves challenging, necessitating reliance on histopathological examination for definitive diagnosis). Despite the possible confounding factors represented by the lichenoid infiltrate characterizing both disorders, epidermal thinning, subepidermal hyalinization and loss of elastic fibers are very peculiar of OLS and are generally absent in oral lichen planus.

A study conducted by Attilli et al. delved deeper into the histopathological characteristics of lip OLS lesions and compared them to those of genital LS. Among the 27 lesions confirmed through histological examination, fifteen were categorized as early inflammatory or presclerotic, eight as intermediate/progressive, and four as late resolved lesions (13). Interestingly, only 44% of the lesions exhibited visible dermal sclerosis confined to the papillary layer, highlighting a distinct histological pattern compared to genital LS lesions. The same group, In genital LS lesions, only 12% were asymptomatic, and a significant majority (69%) displayed both papillary and reticular dermal sclerosis, indicating potential differences in disease progression and manifestation between oral and genital presentations of LS.

### Differential diagnosis

Although OLS is a relatively rare condition, its consideration in the differential diagnosis is crucial when encountering porcelain- or ivory-like whitish macules in the oral cavity.

Particularly, differentiating between OLS and lichen planus, along with other disorders of the oral cavity, is essential given their overlapping clinical features (16–18). Lichen planus and LS share various common characteristics, including lymphocytic infiltration at the dermal-epidermal junction, clinical involvement of both skin and mucosa, erosive mucosal surface disease, and the potential development of squamous cell carcinoma in chronic, erosive mucosal lesions (17). Despite these shared features, documented cases of patients concurrently presenting with evidence of both diseases are notably scarce.

Another possible differential diagnosis is vitiligo, which sometimes accompanies LS. The concept of “Vitiligoid LS,” proposed by Borda et al, introduces a superficial variant that is particularly relevant to lip LS (19). The importance of differentiating this variant resides in the unknown potential of evolution into dysplasia and/or malignancy (16).

Lastly, other differentials include: leukoplakia, oral candidiasis, morsicatio mucosae oris, pemphigus vulgaris, cicatricial pemphigoid (20).

Due to the lack of specific clinical characteristics, biopsy is generally mandatory for the diagnosis of OLS. We therefore recommend histopathological evaluation of all whitish macules and/or plaques of the oral cavity, unless clear patterns of lichen planus (e.g., Wickham striae) or signs of thrush are present.

### Treatment strategies

In general, treatment for OLS was historically not considered necessary unless significant symptoms or aesthetic concerns arise (15, 21). However, due to the scarcity of cases and the elusive nature of treatment recommendations for OLS, management approaches remain a subject of ongoing exploration. Topical or intralesional corticosteroids are typically regarded as the first-line therapy for addressing symptomatic oral lesions in OLS (14, 22). With regards to intralesional corticosteroids, some authors suggest the use of triamcinolone acetonide administered at intervals of 1 or 2 months (4). Topical calcineurin inhibitors are also commonly prescribed as possible therapies for managing oral LS (4, 22).

For smaller lesions, surgical excision has demonstrated efficacy as a standalone therapy, while larger lesions may necessitate the addition of intralesional triamcinolone or corticosteroid injections for effective resolution (14). One patient has been reported to be successfully treated with a free gingival graft in order to restore gingival integrity, highlighting a potential surgical intervention for advanced cases of OLS (23). Other therapeutic options for addressing OLS include topical testosterone and oral colchicine, although further research is warranted to ascertain their efficacy and safety profiles in this context (4, 21).

Guidelines for the management of OLS are currently not available. A systematic review of the published cases of OLS was performed in 2018 by Matela and collaborators, who also described medication options and their efficacy in oral LS (14). The authors could not define established treatment guidelines for OLS, since current evidence was limited to individual case reports. Despite acknowledging that current data do not allow recommendations based on high-level evidence, we agree that first-line treatments could include excision of small lesions and the use of topical potent-ultrapotent corticosteroid ointments or intralesional injections with moderate-potency corticosteroids, such as triamcinolone acetonide. In case of ineffectiveness of these first-line strategies, topical calcineurin inhibitors (e.g., tacrolimus and pimecrolimus) may be considered. Nonetheless, more consistent data on treatment efficacy and duration are needed to develop standardized guidelines for the management of OLS.

### Follow-up and complications

Albeit there have been no reported cases of malignant transformation associated with OLS thus far, it is imperative to emphasize the importance of regular and long-term monitoring of patients diagnosed with this condition (15, 18). Despite its relatively benign nature, continued surveillance is essential to promptly identify any potential complications or changes in lesion morphology that may warrant further investigation.

While the progression of untreated genital LS may lead to scarring and an elevated risk of squamous cell carcinoma, the risk of malignant transformation in OLS remains undocumented (22). Nevertheless, the rarity of OLS presentations in the oral cavity underscores the critical role of routine dental examinations in detecting these lesions early on, facilitating timely intervention and management. In instances where lichenoid changes are observed in oral lesions of patients diagnosed with LS, intensified follow-up protocols are recommended (15, 16). This proactive approach is essential due to the uncertain risk of oral dysplasia in individuals with LS-related oral manifestations (13). Close monitoring and regular assessments enable clinicians to monitor disease progression, identify any potential signs of dysplastic changes, and implement appropriate interventions as needed, thus optimizing patient outcomes and minimizing the risk of adverse outcomes.

However, due to the rarity of the disorder and the absence of long-term follow-up data, clear follow-up practical recommendations did not emerge from literature revision. The paucity of information on OLS-related evolution, however, suggests that even asymptomatic forms of the disease should probably be treated. We also recommend performing a second biopsy (even in case of previous histological confirmation) on OLS lesions not responding to topical therapy and/or developing erosions to rule out underlying malignancies.

## Conclusions

The asymptomatic nature and rarity of OLS pose significant challenges to its recognition and diagnosis within clinical practice. The lack of characteristic symptoms coupled with its infrequent occurrence may contribute to its under-recognition and misdiagnosis, underscoring the importance of heightened vigilance and awareness among healthcare professionals. Meticulous evaluation for the coexistence or absence of oral and genital or cutaneous lesions emerge as indispensable components in advancing our understanding of OLS. Histological confirmation is mandatory to confirm the diagnosis of OLS.

In contrast to its genital counterpart, OLS not only exhibits a less symptomatic and destructive clinical course but is also histologically often characterized by limited dermal sclerosis. To date, no cases of malignant transformation of OLS have been documented. However, most authors agree that patients should undergo regular follow-up due to the scarcity of data in this setting.

This review highlights the critical need for integrating clinical and histopathological findings to improve diagnostic accuracy and optimize treatment strategies for OLS. Such an integrated approach is essential to distinguish OLS from other similar conditions accurately and to ensure appropriate patient management. Future research should prioritize elucidating the underlying pathogenesis of OLS, which remains poorly understood. Investigating the genetic, immunological, and environmental factors that contribute to its development could provide valuable insights. Additionally, exploring novel therapeutic approaches is necessary to address the limitations of current treatments, aiming for more effective and long-lasting solutions.

Assessing the long-term outcomes of patients with OLS is another vital area of research. Longitudinal studies can help determine the natural course of the disease, identify potential complications, and evaluate the effectiveness of various treatment modalities over time. Furthermore, increasing awareness among healthcare providers is crucial for the early detection and effective management of OLS. Educational initiatives and training programs can help clinicians recognize the subtle clinical features of OLS and understand the importance of histopathological confirmation. By enhancing awareness and knowledge, healthcare providers can ensure timely diagnosis, prevent misdiagnosis, and implement appropriate interventions to improve patient outcomes.
